# Qualitative Exploration of Cross-Sector Perspectives on the Contributions of Local Health Departments in Land-Use and Transportation Policy

**DOI:** 10.5888/pcd14.170226

**Published:** 2017-11-22

**Authors:** Meera Sreedhara, Karin Valentine Goins, Semra A. Aytur, Rodney Lyn, Jay E. Maddock, Robin Riessman, Thomas L. Schmid, Heather Wooten, Stephenie C. Lemon

**Affiliations:** 1 University of Massachusetts Medical School, Worcester, Massachusetts; 2 University of New Hampshire, Durham, New Hampshire; 3 Georgia State University, Atlanta, Georgia; 4 Texas A&M University, College Station, Texas; 5 University of Massachusetts Amherst, Amherst, Massachusetts; 6 Centers for Disease Control and Prevention, Atlanta, Georgia; 7 ChangeLab Solutions, Oakland, California

## Abstract

**Introduction:**

Transportation and land-use policies can affect the physical activity of populations. Local health departments (LHDs) are encouraged to participate in built-environment policy processes, which are outside their traditional expertise. Cross-sector collaborations are needed, yet stakeholders’ perceptions of LHD involvement are not well understood. The objective of this study was to describe the perceived value of LHD participation in transportation and land-use decision making and potential contributions to these processes among stakeholders.

**Methods:**

We analyzed qualitative data from 49 semistructured interviews in 2015. Participants were professionals in 13 US states and 4 disciplines: land-use planning (n = 13), transportation/public works (n = 11), public health (n = 19), and other (municipal administration and bike and pedestrian advocacy [n = 6]). Two analysts conducted directed content analysis.

**Results:**

All respondents reported that LHDs offer valuable contributions to transportation and land-use policy processes. They identified 7 contributions (interrater agreement 91%): 1) physical activity and health perspective (n = 44), 2) data analysis and assessment (n = 41), 3) partnerships in the community and across sectors (n = 35), 4) public education (n = 27), 5) knowledge of the public health evidence base and best practices (n = 23), 6) resource support (eg, grant writing, technical assistance) (n = 20), and 7) health equity (n = 8).

**Conclusion:**

LHDs can leverage their strengths to foster cross-sector collaborations that promote physical activity opportunities in communities. Our results will inform development of sustainable capacity-building models for LHD involvement in built-environment decision making.

## Introduction

Active transportation through walking and biking is critical to physical activity promotion (1). Transportation and land-use policies affect the built environment and can enhance active transportation opportunities (2). The responsibility for setting these policies resides with state, regional, and local governing institutions (3), principally with land-use planners and transportation/public works professionals (4). Local health departments (LHDs) (any municipal, county, regional, or network-based public health entity) are encouraged to participate by public health authorities in these policy processes (5), but these processes are outside traditional LHD expertise (6).

LHD collaboration on developing built-environment policy is consistent with a new model of public health practice, Public Health 3.0, which focuses on social and environmental determinants of health through cross-sector collaborations (7). Yet LHD officials are less likely than other municipal officials to engage in transportation and land-use decision making (6). Barriers and facilitators to engagement have been studied (6,8–10), but not contributions made by LHDs to these policy processes. Studies indicate the legal basis for and the value of public health engagement in planning and zoning processes (11), but to our knowledge no studies have examined cross-collaboration to promote community physical activity opportunities. Although planning and zoning have roots in public health, health and transportation is a newer association and largely unexplored (8,12).

It is critical to prioritize strategic approaches to LHD collaboration with land-use planning and transportation/public works agencies (13). A first step is understanding how stakeholders in relevant sectors characterize LHDs’ potential role in these policy processes to establish actionable content areas and points of entry. The objective of this qualitative study was to explore the perceived value of LHD participation in built-environment decision making and the potential role of LHDs in these processes among diverse stakeholders.

## Methods

We conducted semistructured interviews to better understand the potential roles and responsibilities for LHDs in municipal land-use and transportation policy processes. Understanding of relevant policy process was grounded in the research team’s previous work: policy participation (development, adoption, or implementation of municipal land-use policy to increase mixed use, density, street connectivity or pedestrian or bicycle access or municipal public works or transportation policy to increase pedestrian or bicycle safety or accommodation) and policy type (ordinance or bylaw, plan, design standards, reallocation of existing funding, or new funding) (6). Our target sample consisted of state and local professionals who represented land-use planning, economic development, transportation/public works, public health, municipal administration, and active transportation advocacy and had experience in incorporating public health into transportation and land-use processes. Purposive and snowball sampling approaches were used. We identified an initial sample through academic experts in the Physical Activity Policy Research Network Plus (PAPRN+), which is funded by the Centers for Disease Control and Prevention and whose mission is to advance policy research to improve strategies for translation, dissemination, and implementation of policy systems and environmental strategies and increase the number of Americans who achieve adequate levels of physical activity. PAPRN+ colleagues enabled us to invite a wide range of professionals involved in active transportation and health, for which no single list or database exists. We emailed invitations to 62 people. We conducted 2 to 4 interviews with participants in 13 states to achieve diversity by geography, discipline, and community type (population size and economic profile). This study was approved by the institutional review board of the University of Massachusetts Medical School.

### Instrument and measures

A semistructured interview guide was developed by 2 authors (K.V.G. and S.C.L.). Twelve questions asked about respondents’ experiences in working with personnel in other departments of interest, their perspectives on the potential value and contributions of LHD officials to municipal built-environment policy, and their views on the knowledge and skills LHDs need to participate in decision making. Interview questions were iteratively refined through feedback from a research team member (J.E.M.) and pilot interviews conducted with 2 nonparticipating land-use planners. Our analysis focused on 3 questions pertinent to the research objective:

**Question 1.** “Have you personally worked with local or state public health officials?” (asked of all disciplines except public health) or “Have you personally worked with local or state land use or transportation officials?” (asked of public health respondents). If the answer was yes, the respondent was asked, “Would you describe that relationship?” and “What were the strengths or positives of that relationship?”

**Question 2.** “How can LHDs help other municipal departments meet their own goals?”

**Question 3.** “What unique contributions do you think LHDs can make to land use [transportation] policy processes at the local level to increase walkability and bike accommodation, beyond what planners and public works officials do?”

### Data collection and analysis

Interviews took place from May through October 2015. Verbal consent was obtained before each interview. One study team member (K.V.G.) conducted telephone interviews that lasted approximately 1 hour. During the consent process, participants were offered a $50 gift card for their involvement. Interviews were audio recorded and transcribed. The interviewer reviewed all transcripts to remove personal identifiers and confirm accuracy against audio recordings and verbatim notes taken during the interviews.

Qualitative analysis was guided by deductive approach and an adapted version of the coding method suggested by Campbell and colleagues (14,15). We used a start-list approach in which interview questions were used as an initial guide to develop the codebook composed of 3 initial codes: strengths of working relationship, how LHDs can help other departments meet their goals, and unique contributions of LHDs. We found 10 emergent themes: 1) collaboration/partnerships/community engagement, 2) best practices for incorporating public health measures, 3) data, 4) education, 5) public support/input, 6) built environment–health connection, 7) funding, 8) focus on equity, 9) utilization of facilities, and 10) rationale for infrastructure. We then consolidated these themes into 7 mutually exclusive codes (14). Participants’ perceptions of the value of LHD participation in policy processes were first assessed, followed by an assessment of areas with potential for LHD contributions. One author (M.S.) applied codes to all transcripts, and another (K.V.G.) reviewed all applied codes. We calculated percentage agreement on themes to assess interrater agreement (mean, 91%) and discussed disagreements until consensus was achieved. The number of participants who reported each theme at least once was summed. We qualitatively mapped the themes to Public Health 3.0 recommendations. Themes were also sorted by participant discipline (land-use planning, transportation/public works, public health, other), and we excerpted a sample quotation for each theme by discipline. Transcripts were organized and coded using NVivo version 10 (QSR International, 2012).

## Results

Forty-nine respondents from 13 states participated. Overall response rate was 79% (49 of 62); response rate varied by discipline (Table 1). There were no active refusals. Participants represented all targeted disciplines: public health (n = 19), land-use planning (n = 13), transportation/public works (n = 11), and other (municipal administration and bike and pedestrian advocacy) (n = 6). Approximately 60% of participants represented city (n = 19), county (n = 9) or city–county (n = 1) jurisdictions, with the remainder having broader jurisdictions (Table 1).

**Table 1:** Characteristics of Participants^a^ and Nonrespondents in Qualitative Study of Cross-Sector Perspectives on the Contributions of Local Health Departments in Land-Use and Transportation Policy, 2015

Characteristics	No. (%)^b^		
	Contacts (N = 62)	Participants (N = 49)	Nonrespondents (N = 13)
**Discipline**			
Public health	20 (32.3)	19 (38.8)	1 (7.7)
Land-use planning	18 (29.0)	13 (26.5)	5 (38.5)
Transportation and public works	18 (29.0)	11 (22.5)	7 (53.8)
Other^c^	6 (9.7)	6 (12.2)	0
**Jurisdiction**			
City or town	26 (41.9)	19 (38.8)	7 (53.8)
City–county	1 (1.6)	1 (2.0)	0
County	11 (17.7)	9 (18.4)	2 (15.4)
State	10 (16.1)	9 (18.4)	1 (7.7)
Region	9 (14.5)	6 (12.2)	3 (23.1)
National	2 (3.2)	2 (4.1)	0
Not a jurisdiction (university)	3 (4.8)	3 (6.1)	0
**Organization type**			
Administration	3 (4.8)	3 (6.1)	0
Advocacy or capacity-building organization	10 (16.1)	9 (18.4)	1 (7.7)
Consulting	2 (3.2)	2 (4.1)	0
Health department	14 (22.6)	13 (26.5)	1 (7.7)
Planning/community or economic development department	12 (19.4)	9 (18.4)	3 (23.1)
Regional planning agency or Metropolitan Planning Organization	6 (9.7)	3 (6.1)	3 (23.1)
Transportation or public works department	12 (19.4)	7 (14.3)	5 (38.5)
University	3 (4.8)	3 (6.1)	0

a Respondents represented 13 states: Arizona, Florida, Georgia, Illinois, Kansas, Massachusetts, Minnesota, Mississippi, New York, Oregon, Tennessee, Washington, and Wisconsin.

b Percentages may not total 100 because of rounding.

c Other disciplines include municipal administration and bike and pedestrian advocacy.

### Themes

All respondents uniformly reported that LHDs had potentially valuable contributions to built-environment policy processes. One planner mentioned being a “big proponent of multidisciplinary approaches to decision making” because “you end up with . . . a better product if you have all of the players at the table and involved in each of those processes.” A transportation/public works professional who agreed stated that “local health folks can make a contribution by guiding work that’s community based . . . continuing to help people think about transportation and the designing and building of roads as beyond ‘the road starts here and it ends here.’”

We identified 7 themes for the potential contributions of LHDs to land-use and transportation policy processes and aligned them with Public Health 3.0 recommendations (Table 2). We selected 27 sample comments (Table 3). Although the interview guide asked separate questions about land use and transportation, public health respondents did not express different views about working with land-use planners versus transportation personnel, and non–public health respondents did not express different views about the capacity of public health personnel to collaborate on land-use planning versus transportation. 

**Table 2:** Seven Qualitative Themes Ranked by Frequency of Mention by Study Participants and How They Align With Public Health 3.0 Recommendations, Qualitative Study of Cross-Sector Perspectives on the Contributions of Local Health Departments in Land-Use and Transportation Policy, 2015

Theme	No. of Participants Who Mentioned Theme	Public Health 3.0 Recommendations
Physical activity and health perspective	44	Strong leadership and workforce
Data analysis and assessment	41	Timely and locally relevant data, metrics, and analytics
Partnerships	35	Strategic partnerships
Public education	27	Strong leadership and workforce; Foundational infrastructure
Knowledge of evidence base and best practices	23	Strong leadership and workforce; Foundational infrastructure
Resource support	20	Strong leadership and workforce; Flexible and sustainable funding; Foundational infrastructure
Health equity	8	Strong leadership and workforce; Strategic partnerships; Foundational infrastructure

**Table 3:** Sample Comments by Participants and Number of Participants Who Mentioned Theme, by Discipline of Participants and Theme, in Qualitative Study of Cross-Sector Perspectives on the Contributions of Local Health Departments in Land-Use and Transportation Policy, 2015

Land-Use Planning (n = 13)	Transportation/Public Works (n = 11)	Public Health (n = 19)	Other^a^ (n = 6)
**Theme: Physical Activity and Health Perspective**			
n = 12	n = 10	n = 16	n = 6
[H]aving a public health perspective broadens the conversation and I’ve found it’s gotten people to take notice. When we first started to do community engagement, we didn’t get a lot of people when we were talking about brownfields in a technical way. When we shifted the conversation to talk about public health and started holding meetings at the local hospital, people got more interested and that group probably grew from five people to thirty — just by using that language.	I think it takes a lot of effort and education to create policy — to get politicians to create different policies, and you need a large support base. So I think health departments could set the foundation and groundwork for establishing or getting established the policy that we need.	[W]hen you put a health framework on it, it helps residents and people become more engaged and more invested in the process and understand why it matters.	[I]t also has broadened us as a movement, transportation folks — getting us outside of our box a little bit in terms of roads and streets and fighting with the local traffic engineer to build a sidewalk. We’ve figured out a different way to talk about it.
**Theme: Data Analysis and Assessment**			
n = 10	n = 10	n = 17	n = 4
What would further that conversation at the local level and the policy level, especially among the decision makers is to have better data, more localized data of what the actual obesity rate is and being able to match that up with areas that don’t have any sidewalks or trails — the roads are not safe, they don’t provide a space for bicycles or pedestrians — access to transit.	A lot of this is based on political will, as far as the direction that we go in, and if there’s information out there that the health department can provide that would show, or more substantiate the benefit and utilization of these types of facilities, the more information we have, the more data we have, it helps us when we’re trying to prioritize things throughout the county.	They have a lot of access to data. Health statistics, you know, where the cities can bring the crash data and all of that, health departments can bring BMI and heart . . . chronic disease rates, or how many people are getting the physical activity . . . all of that BRFSS information.	The health departments understanding that, and then facilitating and saying to the other departments or boards “here is something that can be utilized to help all of us make better decisions for the health of the community.”
**Theme: Partnerships**			
n = 7	n = 7	n = 16	n = 5
[B]roadening the outreach through different partners and public health in particular does have a lot of fingers out there.	[T]here’s been a very significant push by local health agencies over the last ten years here, in this local area, bringing together partnerships. I think that’s had a lot of crossbreeding benefits. It’s helped planners become more familiar with the different agencies that are out there and what the emphasis is for each agency. It’s certainly brought together coalitions where we never saw them before.	[I]f you can show how cross-cutting all of these issues are, it gives it a little bit more depth, so it’s not just DPW going in and saying, “Well, we really need to do this.” It’s other experts in the field. I think that our role is to help assemble those people and get them to the table to help inform with data.	Working with public health gave transportation advocates a whole different set of leverage points and relationships with not just public health people, but people in a community that were being served by the public health agencies, who we wouldn’t normally interact with in our somewhat closed transportation world. So I think it really expanded the universe of people and groups that I was able to work with.
**Theme: Public Education**			
n = 7	n = 4	n = 13	n = 3
They could really help with that public education piece in terms of pointing out why certain land use or certain transportation actions or focuses are so important from a public health standpoint. That’s something that planners and to an even greater degree engineers aren’t particularly good at, but public health officials could more effectively make that argument.	Being that interface with the community to say . . . to help with the stakeholder involvement.	[T]he city planning department and even the public works department, they’re not, in their role, they can’t spend time cultivating the champions of the community and so I think that’s, you know, an appropriate place for us to be. And I imagine that most health departments historically probably have more experience trying to identify and support advocates.	A lot of times when boards are trying to adopt health policies there’s some pushback from the public and if the public were indoctrinated ahead of time by the state health department or the LHD it would make our job at the local governance level a lot easier.
**Theme: Knowledge of Evidence Base and Best Practices**			
n = 11	n = 4	n = 6	n = 2
[P]lanners will incorporate public health concepts to the level they know them, but I don’t think they’ll know them as well as public health experts. So that’s where I think you need public health experts to be involved, but not necessarily to be involved from A to Z. It might be they’re involved from A to L, and then let planners do the M to Z portion while public health folks start focusing on other public health concepts.	[I]f it’s their voice at the table that’s saying “this project will have these benefits,” I think that carries a lot more weight than if it’s an engineer or even a planner saying: “here’s what we think the benefits are.”	[They] provide the science, the evidence-based science, behind why this is an important public health initiative.	[T]he public health agencies have been really the key state and local agencies that were willing to fund doing training and assessments and really getting people thinking about these issues in communities. I think that stems from both the long tradition of saying “we need people to get more exercise so they’re healthier,” but the more recent realizations say, within the last decade, that it just isn't enough to tell people they need to get more exercise. We need to make it possible and safe and convenient for them to go out and get more exercise. So if it’s not safe to bike or walk, or if there’s no place for them to bike or walk in their communities, they’re just not going to do it.
**Theme: Resource Support**			
n = 4	n = 2	n = 11	n = 3
If we had more people from that [health] side coming to testify, coming to participate on behalf of the kinds of things that we are doing — the transition that we’re trying to make from being an auto-dominated suburb to a place where walking is a priority, pedestrians are a priority — the health and social life of our residents — something that we care about. If it weren’t just us carrying a message of human health, but actual health practitioners here also singing that song that would allow us to power through some of the troubles that we have.	[A health director] was very visionary in that and brought us in — what can we do to augment some of the resources that were limited in certain ways because some of the grants that were coming in.	We have two analysts . . . and a GIS person, that I think, the three of them could easily be brought into discussions about how various developments might impact health. And so, I think we’re kind of, in that sense, maybe an underutilized resource that perhaps if they just understood and developed more relationships with us, then that would become a sort of an extra resource for them.	The opportunity to leverage funding is always a benefit. I know whenever we apply for grant funding they’re looking for local commitment, local match or in-kind investment, and so I think when you have multiple agencies working together, limited resources are able to be expanded beyond what you’d be able to do by yourself so there’s some synergy there when two or more organizations are working together in terms of being able to do more with less.
**Theme: Health Equity**			
n = 1	n = 3	n = 4	n = 0
The potential impact that they can have on helping people have more access to opportunities that would keep them healthier and access to opportunities that the literature says this is what’s good for people. I found when you open up that sense of possibility for people, you can generate a level of excitement in the room that the people are really looking for.	We put low-income housing at a place that had very poor transportation access for people who mostly didn’t own cars. And so that [relationship with LHD] really in my mind brought forward this issue of transportation equity. All things that I normally wouldn’t have thought about.	[W]e can contribute by bringing the voices to the table that aren’t often represented in the political process. And, you know, if they can’t be engaged, at least being a proxy for those . . . the needs of those tacit users.	(No quotes coded for this discipline.)

a Other disciplines include municipal administration and bike and pedestrian advocacy.

### Physical activity and health perspective

Participants frequently (n = 44) described the emphasis that LHDs placed on health impacts of the built environment as a valuable contribution in the local built-environment policy process. This theme was also woven throughout each of the other themes. One public health official (Participant A) stated that “health was not something that was part of any conversation. . . . That’s what I brought to these groups and that they developed an appreciation for it and came to understand the importance of it and how it impacted the community.” This comment echoed the sentiment of others that the health perspective is germane to broadening the appeal and importance of transportation and land-use discussions. Participants indicated that LHDs may help generate synergy among transportation-related goals and priorities of different departments. Some respondents in land-use planning suggested that LHDs can help incorporate these goals into existing priorities.

### Data and assessment

LHDs’ role in accessing, collecting, analyzing, and collaborating on health data and assessments was frequently cited (n = 41). Access to data (eg, community health assessments, community health profiles, local data sets) may serve as a supplement for planners, transportation/public works or other non–public health disciplines. One transportation official (Participant B) said, “[I]f there’s information out there that the health department can provide that would show, or more substantiate the benefit and utilization of these types of facilities . . . the more data we have, it helps us when we’re trying to prioritize things throughout the county.” A public health participant (Participant C) described having staff “with the technical skills in terms of our assessment function to identify the relationships between . . . health and land use and transportation.” Participants indicated that LHDs may be able to gather qualitative community-level data to shed light on barriers to active transportation. Relationships between LHDs and academic and analytic partners were also named as valuable.

### Partnerships

Partnerships that LHDs engage in was another commonly reported contribution (n = 35). Respondents in each discipline spoke of the ability of LHDs to build and develop new relationships among community partners and municipal departments. Public health professionals described the discipline’s tradition of bringing stakeholders together and reported the desire to collaborate on built-environment policy. LHDs were perceived as able to engage with diverse partners (community members, advocates, academia, external departments, and other sectors). Perceived impacts of partnerships were related to other themes (physical activity and health perspective and resource support). One transportation professional (Participant D) summed the theme by saying, “[T]his web of relationships and impacts is big, broad, and lovely.”

### Public education

Respondents viewed LHDs as able to engage the public, thereby enhancing public support or involvement in transportation and land-use policies and projects, often through educating the community on the health benefits of policies (n = 27). LHDs were described as engaging the public in numerous ways, including holding meetings with community members, communicating and translating complex information (eg, average daily traffic, transportation impact fee), developing trust through ongoing presence and engagement, and serving as advocates. Additionally, LHDs may assist in relaying information on concerns, barriers, and use of active transportation because of their relationship with the public. One planner (Participant E) mentioned LHDs’ role in “raising public awareness and promoting discussion among the general population so they can better understand the link between the built environment and their health.” One public health official (Participant F) described the LHD role as “softening the ground and working with the community and framing department of transportation changes with a health lens.”

### Knowledge of evidence base and best practices

LHDs’ knowledge of the evidence base and best practices (n = 23) in public health and active transportation was also reported. LHDs may be able to contribute to their own communities’ information on the evidence base and best practices from other communities. As such, their voices may carry weight in land-use and transportation discussions. One land-use planner (Participant G) indicated that the knowledge of LHDs could lead to “fuller discussion of the reasons why we might be advocating for mixed-use neighborhoods” by framing conversations dominated by local concerns in a way that may help planners experiencing pushback from community members on transportation initiatives. A respondent in transportation/public works (Participant H) described public health officials as being able to help focus transportation programming by consistently asking, “[I]s that an evidence-based project?”

### Resource support

Twenty respondents described various dimensions of LHDs’ potential to provide support to built-environment policy and processes; the most common dimension cited was providing assistance with grant proposals and leveraging resources. Participants suggested tapping LHDs to help identify, write, secure, and leverage grants. As both a land-use planner and a health official noted, LHDs may also be a source of funding for small signage grants as well as the administration of funds for bike and pedestrian grant programs. Incorporating the LHD into funding applications was viewed as beneficial as it “lends credence,” and funders look positively on applications that include local commitment, local matching grants, or in-kind investment.

### Health equity

Less frequently cited was LHDs’ ability to focus a health-equity lens on the transportation and land-use policy process (n = 8). Respondents indicated that LHDs can draw attention to the health impact of land-use and transportation decisions on vulnerable populations, which complements the environmental justice lens used in planning and transportation. One transportation/public works (Participant I) respondent recalled the relationship with the LHD as what “brought forward this issue of transportation equity,” which otherwise would have been overlooked. Participants explained that LHDs’ traditional role as advocates for the community and especially underserved populations may allow them to serve as a proxy for the underserved when transportation and land-use decisions are being made.

## Discussion

This qualitative study confirmed that stakeholders from a range of disciplines perceive value in LHD participation in local land-use and transportation policy processes. Seven potential LHD contributions that build from common LHD strengths were identified: physical activity and health perspective, data analysis and assessment, partnerships, public education, knowledge of evidence base and best practices, resource support, and health equity.

To our knowledge, ours is the first US study to explore the perceived value of LHD participation on built-environment decision making among diverse stakeholders. The 7 themes of potential contributions of LHDs to built-environment policy processes identified mirror core public health values, capabilities and functions promulgated by national public health leaders such as the American Public Health Association (APHA) (16) and the Institute of Medicine (17) in the past 3 decades. The contributions identified in our study may represent content areas through which LHDs can fulfill core public health functions and Public Health 3.0 efforts in transportation and land-use policy decision making (Table 2) (7).

Our findings are particularly relevant given that interest in cross-sector collaboration to address complicated problems is growing across many disciplines as stakeholders acknowledge the need to move away from “vertically organized” (ie, siloed) work (18). Discussion about making communities more walkable and bikeable is no exception. Strategic approaches to cross-sector collaboration on active transportation must consider additional factors, such as economic and environmental benefits (19–21). Multiple co-benefits suggest many potential partners for increasing walking and bicycling opportunities, only some of whom have direct responsibility for the built environment. In 2014, the Centers for Disease Control and Prevention funded a partnership of the American Planning Association and APHA that in turn made grants to 18 Plan4Health community-level partnerships across the United States in which planners work closely with their public health counterparts (22). Four metropolitan planning organizations are models of how transportation agencies are incorporating public health into transportation planning (23). Systematic efforts to build the capacity of health organizations to engage in such collaboration are scarce, however, with limited attempts to conceptualize the public health role in cross-sector collaboration to increase active transportation (24–26) or explore enhancement of LHDs’ capacity to engage (8,27). Our study provides a starting point for strategic approaches to enhancing such collaborations. In addition, methods and results from this study could be applied to establish points of entry for collaboration at the local level in other areas.

Our study focused on the value, or positive benefit, of LHD involvement in built environment processes as leverage. Study participants also identified challenges, or barriers, to this involvement, including resources/capacity, lack of built-environment content expertise among public health officials, language and cultural gaps between the professions, separation or isolation of public health from other municipal functions, and politics and leadership, but these challenges were not the focus of our study. Of these barriers, cultural gaps, resources, and politics were cited most frequently by public health respondents.

Our study has strengths and limitations. It aimed to enroll a purposeful, national sample by employing a snowball sampling method. However, the sample is not nationally representative because participants were selected nonrandomly. Selection bias is possible given that respondents were recruited for their experience in incorporating public health into transportation or land-use processes. Most were also referred through relationships with institutions participating in PAPRN+, which could indicate they see more value in public health collaborations than peers without those relationships. Findings should be interpreted cautiously, as respondents from non–public health disciplines may be more sanguine than nonrespondent peers about the value of collaboration with public health. The sample size precluded quantitative bivariate analysis, but it is relatively large for qualitative research. Although a member-checking process was not used, and a second analyst reviewed applied codes rather than independently applying them, our coding approach resulted in a high interrater agreement, which helps support the reliability of our findings.

Our study identified 7 conceptually consistent themes that represent opportunities by which LHDs can leverage their strengths to foster cross-sector collaborations that promote opportunities for active transportation in communities. Results from our study and our continuing work will inform the development of standards of involvement and capabilities of LHDs to engage in transportation and land-use planning decision making. Because responses were similar for both policy areas, we opted to develop a single, unified set of capabilities for ease of use in the field. Furthermore, our findings will inform the development of sustainable capacity-building strategies that increase the skills, infrastructure, and resources of LHDs to engage in built-environment decision making to help achieve active community environments.

## References

[1] Celis-Morales CA , Lyall DM , Welsh P , Anderson J , Steell L , Guo Y , Association between active commuting and incident cardiovascular disease, cancer, and mortality: prospective cohort study. BMJ 2017;357:j1456. 10.1136/bmj.j1456 28424154

[2] Guide to Community Preventive Services. Physical activity: built environment approaches combining transportation system interventions with land use and environmental design. 2016. https://www.thecommunityguide.org/findings/physical-activity-built-environment-approaches. Accessed July 21, 2016.

[3] Transportation Research Board. Does the built environment influence physical activity?: Examining the evidence. Special report no. 282. 2005. www.TRB.org/publications/sr/sr282.pdf. Accessed July 21, 2016.

[4] National Center for O*NET Development. Details report for urban and regional planners (19-3051.00). https://www.onetonline.org/link/details/19-3051.00. Accessed March 12, 2017.

[5] American Public Health Association. Creating policies on land use and transportation systems that promote public health. Policy statement no. 20044. 2004. https://www.apha.org/policies-and-advocacy/public-health-policy-statements/policy-database/2014/07/02/14/17/creating-policies-on-land-use-and-transportation-systems-that-promote-public-health. Accessed July 21, 2016.

[6] Lemon SC , Goins KV , Schneider KL , Brownson RC , Valko CA , Evenson KR , Municipal officials’ participation in built environment policy development in the United States. Am J Health Promot 2015;30(1):42–9. 10.4278/ajhp.131021-QUAN-536 25372234 PMC4759645

[7] US Department of Health Human Services. Public Health 3.0: a call to action to create a 21st century public health infrastructure. 2016. Washington (DC): US Department of Health and Human Services. https://www.healthypeople.gov/sites/default/files/Public-Health-3.0-White-Paper.pdf. Accessed March 30, 2017.

[8] Rube K , Veatch M , Huang K , Sacks R , Lent M , Goldstein GP , Developing built environment programs in local health departments: lessons learned from a nationwide mentoring program. Am J Public Health 2014;104(5):e10–8. 10.2105/AJPH.2013.301863 24625166 PMC3987592

[9] Li J , Casey C , Brewer LK . Exploring opportunities for engaging public health organizations in transportation planning. Public Works Manag Policy 2015;20(3):201–25. 10.1177/1087724X14559520

[10] Goins KV , Schneider KL , Brownson R , Carnoske C , Evenson KR , Eyler A , Municipal officials’ perceived barriers to consideration of physical activity in community design decision making. J Public Health Manag Pract 2013;19(3, Suppl 1):S65–73. 10.1097/PHH.0b013e318284970e 23529058 PMC4928376

[11] Ashe M , Jernigan D , Kline R , Galaz R . Land use planning and the control of alcohol, tobacco, firearms, and fast food restaurants. Am J Public Health 2003;93(9):1404–8. 10.2105/AJPH.93.9.1404 12948952 PMC1447982

[12] Cole R , Burke M , Leslie E , Donald M , Owen N . Perceptions of representatives of public, private, and community sector institutions of the barriers and enablers for physically active transport. Transp Policy 2010;17(6):496–504. 10.1016/j.tranpol.2010.05.003

[13] Teutsch SM , Fielding J . Economics and Local Public Health Departments. Am J Prev Med 2016;50(5, Suppl 1):S81–3. 10.1016/j.amepre.2015.10.011 27102862

[14] Miles MB , Huberman AM . Qualitative data analysis: an expanded sourcebook. Thousand Oaks (CA): Sage Publications; 1994.

[15] Campbell JL , Quincy C , Osserman J , Pedersen OK . Coding in-depth semistructured interviews: problems of unitization and intercoder reliability and agreement. Sociol Methods Res 2013;42(3):294–320. 10.1177/0049124113500475

[16] American Public Health Association. Our values. https://www.apha.org/about-apha/our-values. Accessed February 21, 2017. 2017. 10.2190/NS.20.3.l 20943481

[17] Institute of Medicine. Committee for the Study of the Future of Public Health. The future of public health. Washington (DC): National Academy Press; 1988.

[18] Rapson R . Cross sector collaboration is vital to economic development. Delivered November 13, 2014 at the Boston Federal Reserve Bank. http://kresge.org/library/cross-sector-collaboration-vital-economic-development. Accessed July, 20, 2016.

[19] Leinberger C , Alfonzo M . Walk this way: the economic promise of walkable places in metropolitan Washington (DC): The Brookings Institution; 2012.

[20] Maizlish N , Woodcock J , Co S , Ostro B , Fanai A , Fairley D . Health cobenefits and transportation-related reductions in greenhouse gas emissions in the San Francisco Bay area. Am J Public Health 2013;103(4):703–9. 10.2105/AJPH.2012.300939 23409903 PMC3673232

[21] Wang ML , Goins KV , Anatchkova M , Brownson RC , Evenson K , Maddock J , Priorities of municipal policy makers in relation to physical activity and the built environment: a latent class analysis. J Public Health Manag Pract 2016;22(3):221–30. 10.1097/PHH.0000000000000289 26079657 PMC4677062

[22] American Planning Association. Plan4Health: An American Planning Association Project. www.plan4health.us/. July 21, 2016.

[23] US Department of Transportation. Research and Innovative Technology Administration; John A. Volpe National Transportation Systems Center. Metropolitan Area Transportation Planning for Healthy Communities (FHWA-HEP-13-006). http://www.fhwa.dot.gov/planning/health_in_transportation/resources/healthy_communities/mpohealth12122012.pdf. Accessed on July 21, 2016.

[24] Rudolph L , Caplan J , Ben-Moshe K , Dillon L . Health in all policies: a guide for state and local governments. Washington (DC): American Public Health Association; 2013.

[25] Dyjack DT , Botchwey N , Marziale E . Cross-sectoral workforce development: examining the intersection of public health and community design. J Public Health Manag Pract 2013;19(1):97–9. 10.1097/PHH.0b013e3182788cff 23169410

[26] Botchwey ND , Hobson SE , Dannenberg AL , Mumford KG , Contant CK , McMillan TE , A model curriculum for a course on the built environment and public health: training for an interdisciplinary workforce. Am J Prev Med 2009;36(2, Suppl):S63–71. 10.1016/j.amepre.2008.10.003 19147063

[27] Miro A , Perrotta K , Evans H , Kishchuk NA , Gram C , Stanwick RS , Building the capacity of health authorities to influence land use and transportation planning: lessons learned from the Healthy Canada by Design CLASP Project in British Columbia. Can J Public Health 2014;106(1, Suppl 1):eS40–52. 10.17269/cjph.106.4566 25955547 PMC6972227

